# Draft Genome Sequence of the Saccharomyces cerevisiae *×* Saccharomyces kudriavzevii HA1836 Interspecies Hybrid Yeast

**DOI:** 10.1128/genomeA.00343-18

**Published:** 2018-05-17

**Authors:** Ksenija Lopandic, Hakim Tafer, Katja Sterflinger

**Affiliations:** a VIBT-Extremophile Center, University of Natural Resources and Life Sciences Vienna, Vienna, Austria

## Abstract

Saccharomyces cerevisiae × Saccharomyces kudriavzevii interspecies hybrid yeasts have frequently been isolated from alcoholic fermentation environments. Here, we report the draft genome sequence of the S. cerevisiae × S. kudriavzevii HA1836 strain isolated from grapes from an Austrian vineyard.

## GENOME ANNOUNCEMENT

Saccharomyces cerevisiae × Saccharomyces kudriavzevii hybrids have been identified in environments associated with brewing, wine making, and cider production (1, 2). These hybrids acquired beneficial fermenting properties from the S. cerevisiae species and cryotolerance from the S. kudriavzevii species (3). The S. cerevisiae × S. kudriavzevii hybrids have been isolated primarily from cold fermentations, which are characteristic of European regions with cooler continental and oceanic climates (i.e., Austria, Germany, Switzerland, France, and northern Spain). The hybrid strains studied so far differ in the complexity of their genome structures, the proportion of hybridizing yeasts, and their fermentation performances (3–6). Natural, commercial, and artificially constructed hybrid yeasts have shown that the genomic moiety of S. kudriavzevii is more unstable than that of S. cerevisiae and prone to substantial reduction after a hybridization event (3, 6, 7). S. cerevisiae × S. kudriavzevii hybrids may have strong advantages over their parental strains due to better adaptation to cold fermentation conditions, better production of esters, higher alcohols and glycerol, and an increased fructose/glucose fermentation rate (4, 5, 8, 9).

During a study of the yeast biodiversity of Austrian vineyards, several strains were isolated from grapes that were shown to have a S. cerevisiae × S. kudriavzevii hybrid genome by different genetic and molecular markers (7, 10). In order to explore differences in the genome constitutions and gene regulations among the S. cerevisiae × S. kudriavzevii yeasts, we generated the whole-genome sequence of the HA1836 strain. Genome sequencing was carried out using Ion Torrent technology (Ion PGM Hi-Q View kit; Life Technologies, Inc., Carlsbad, CA, USA) according to the manufacturer’s protocols. A total of 1.62 Gb of read data, with a median read length of 315 bp, was produced and assembled with Newbler version 2.9 into a 22.28-Mb genome (∼70× coverage) containing 979 contigs (*N*_50_, 60,823 bp). Comparative analysis of the genome sequences of the HA1836 and VIN7 (11) strains showed a coverage of 19.51 Mb (87.5%).

### Accession number(s).

The whole-genome shotgun project reported here has been deposited at DDBJ/EMBL/GenBank under the accession number PQXS00000000. The version described in this paper is the first version, PQXS01000000.
